# To shift or not to shift: identifying and correcting patient motion after couch rotations in non‐coplanar intracranial radiosurgery with stereoscopic X‐ray imaging

**DOI:** 10.1002/acm2.70505

**Published:** 2026-03-08

**Authors:** Vanessa Da Silva Mendes, Sylvia Garny, Lili Huang, Stephan Schönecker, Christian Trapp, Frederik Fuchs, Christopher Kurz, Claus Belka, Guillaume Landry, Michael Reiner, Stefanie Corradini

**Affiliations:** ^1^ Department of Radiation Oncology LMU University Hospital Munich Bavaria Germany; ^2^ German Cancer Consortium (DKTK) partner site Munich a partnership between DKFZ and LMU University Hospital Munich Bavaria Germany; ^3^ Bavarian Cancer Research Centre (BZKF) Munich Bavaria Germany

**Keywords:** IGRT, intracranial, non‐coplanar beams, patient (re‐)positioning, SRS, stereoscopic X‐ray imaging, X‐ray intrafraction monitoring

## Abstract

**Background:**

Frameless linear accelerator (linac)‐based image‐guided stereotactic radiosurgery (SRS) or fractionated stereotactic radiotherapy (FSRT) are a widely used treatment option for intracranial lesions. Given the high radiation doses involved, it is crucial to maintain precise patient positioning throughout treatment. This requires that geometric inaccuracies arising from patient motion or setup errors are identified and corrected. With frameless immobilization, image‐guidance has a greater impact, especially in non‐coplanar settings that can lead to patient motion and discrepancies between couch and radiation isocenters.

**Purpose:**

Both patient and phantom studies were conducted to assess and quantify the magnitude of geometric uncertainties after couch rotations, aiming at evaluating the clinical need for their correction to warrant a precise treatment delivery.

**Methods:**

Intrafraction X‐ray data, performed by ExacTrac Dynamic (ETD) to monitor and correct patients’ position throughout treatment delivery, were collected from 50 patients treated for brain metastases in stereotactic non‐coplanar schemes and immobilized by stereotactic double‐layered thermoplastic mask systems: 26 patients treated in 40 single‐fraction SRS (168 stereoscopic X‐ray images); 24 treated with FSRT in 128 fractions (278 stereoscopic X‐ray images). Additionally, a head phantom was utilized and 350 measurements under two different couch loads were carried out to distinguish true patient motion from deviations caused by couch rotations or system‐related effects. For both studies, ETD stereoscopic X‐rays were acquired after each couch rotation and the first measured positioning deviation was calculated by comparing X‐ray images to the treatment plan's digitally reconstructed radiographs.

**Results:**

Clinically relevant deviations were observed, exceeding clinical tolerance (≥ 0.5 mm/0.5°) mostly in the lateral and yaw directions and requiring repositioning in nearly half of the couch rotations. These deviations measuring up to 2 mm, revealed to be emerging mainly from patient motion rather than linac setup, as the phantom study showed maximum deviations of up to 0.6 mm and 0.4° when simulating a patient treatment and an interquartile range that did not exceed 0.2 mm and 0.2°.

**Conclusions:**

These findings demonstrate the importance of a continuous intrafraction motion monitoring and repositioning in cranial stereotactic treatments, especially in non‐coplanar settings.

## 1 INTRODUCTION

In modern radiotherapy (RT), the number of stereotactic treatments for intracranial lesions, either in a single‐fraction, as stereotactic radiosurgery (SRS) or as fractionated stereotactic radiotherapy (FSRT) is still increasing. These techniques bring considerable advantages for the patient, providing high local control rates and having a more favorable impact on quality of life, when compared to whole brain radiation therapy, including a reduced risk of neurocognitive impairment^1^, ^2^

SRS and FSRT are characterized by the delivery of high radiation doses in one or few fractions, with the additional advantage of enabling the treatment of multiple lesions using a single isocenter.^3^ In frameless radiosurgery setups, positioning devices provide rigid motion restriction, enabling the use of small margins to the gross tumor volume (GTV) to better spare surrounding normal tissues.^4^ However, planning target volume (PTV) margins must remain sufficiently large to compensate for residual uncertainties, deriving for instance from image registration, target delineation or an inaccurate isocenter position and known as systematic errors and/or residual random setup errors.^5^, ^6^, ^7^ Van Herk studied the error sources in RT, described methods to quantify the effect of geometrical uncertainties and derived a margin formula, utilising probability distributions of the cumulative dose delivered to the CTV for a population of patients, ensuring that 90% of the patients would receive 95% of the prescribed dose.^8^, ^9^ This calculation was developed in different stages, where firstly the estimated cumulative blurred dose distributions, attained by adding the dose distribution for each fraction, were calculated by performing the convolution of the dose distribution with all random variations, including setup and organ motion.^10^, ^11^, ^12^, ^13^, ^14^ The following stage was to determine the dose distribution in the CTV when applying a certain systematic component to the blurred dose distribution.^11^, ^13^ Moreover, to find out which portion of the population will receive a specific dose distribution, they used the probability distribution of systematic errors.^8^ Nevertheless, it is important to mention that this recipe was developed for multiple‐fraction treatments, and its direct use for single‐fraction treatments would not be appropriate with the assumption of non‐existing average systematic setup errors, or the fact that it is no longer acceptable that part of the CTV would be outside of the radiation field for some fractions due to its single‐fraction nature.^7^ Therefore, it would not provide an accurate estimation of the uncertainties for CTV‐PTV margins.^7^ Single‐fraction treatments present different characteristics and therefore Zhang et al. proposed a mathematical approach to define CTV‐PTV margins for single‐fraction intracranial SRS treatments, where a nonzero systematic error for a specific machine is included.^7^ Yet, clinical experience continues to play a pivotal role in margin selection. Different clinical studies report PTV margins in the range from 0 to 2 mm for SRS of brain metastases^15^, ^16^ without clear consensus and depending on the technology used. In our experience, a 1 mm PTV margin on a frameless linear accelerator (linac) platform has achieved excellent results.^17^

To achieve steep dose gradients and high dose conformity, treatments on a C‐arm linac typically employ multiple non‐coplanar couch angles. Furthermore, for patient positioning and immobilization purposes, non‐invasive frameless devices, such as thermoplastic masks, are currently and widely utilized.^18^, ^19^

Thermoplastic masks are individually moulded for every patient prior to the acquisition of the planning computed tomography (CT) scan. They are applied at every treatment session to ensure reproducible positioning throughout treatment delivery.^20^ Nowadays, non‐invasive thermoplastic masks are preferred over invasive fixation methods because of improved patient comfort. However, they remain less rigid and allow more motion than invasive approaches.^21^ In particular, single‐layer thermoplastic masks allow for more patient movement compared to stereotactic double‐layered systems.^20^ Additionally, long delivery times or even couch rotations, both common in stereotactic cranial treatments, represent further sources of patient motion and need to be taken into account.^19^, ^20^, ^22^

Non‐coplanar couch positions can lead to deviations between couch and radiation isocenters, thereby inducing uncertainties during treatment delivery.^6^, ^19^, ^23^ To minimize patient motion and geometrical error, frequent patient imaging and repositioning throughout the treatment is crucial, and made possible by image‐guided radiotherapy (IGRT) systems.^19^, ^24^ Nevertheless, all uncertainties, other than patient motion, must be investigated in advance through a Winston‐Lutz (WL) test and kept below 1 mm.^25^ This ensures that the imaging and mechanical isocenters coincide with the radiation isocenter within a tolerance of 1 mm.^6^

ExacTrac Dynamic (ETD) (Brainlab SE, Munich, Germany) is an IGRT system that provides real‐time, continuous image guidance by combining X‐ray imaging with optical/surface imaging. This system was installed at our institution in June 2020, and several quality assurance tests were performed to proceed with the commissioning of the system, as well as frequent consistency tests, described in the literature.^26^, ^27^

Several studies have compared and evaluated different positioning systems in both frame‐based and frameless cranial stereotactic radiotherapy and radiosurgery, assessing translational and rotational accuracy, comparing different imaging systems or comparing different types of immobilization thermoplastic masks.^19^, ^20^, ^21^, ^22^, ^23^, ^28^, ^29^, ^30^, ^31^, ^32^, ^33^, ^34^, ^35^, ^36^, ^37^, ^38^, ^39^, ^40^, ^41^ Usually, IGRT is performed at 0° couch angle using cone‐beam CT (CBCT) at the beginning of the treatment, and geometric accuracy within < 1 mm is verified beforehand through a WL test. However, there are limited data on patient deviations in this setting, as most IGRT systems cannot provide intrafraction positioning information at non‐coplanar couch positions.

In our institution, the ETD system is fully implemented throughout the entire cranial treatment workflows, for initial positioning, continuous monitoring and eventually repositioning of patients during treatment delivery when tolerances are exceeded. Our study specifically examined patient deviations after couch rotations during non‐coplanar stereotactic treatments, independent of beam‐on time, as the largest displacements commonly occur directly after couch rotations. This addresses a gap in the literature, as most studies have focused on intrafraction beam‐on deviations. A further goal was to assess the magnitude of these deviations and to evaluate the clinical necessity of correcting them to achieve a highly precise radiosurgery treatment. We used the ETD system to quantify the magnitude of these deviations and to evaluate their clinical impact, particularly whether they exceeded the applied positioning tolerances. For this purpose, we assessed the initial intrafraction position errors for each couch angle, in all 6 degrees of freedom (6DoF), for patients treated with non‐coplanar SRS and FSRT by an Elekta Versa HD linac (Elekta AB, Stockholm, Sweden), equipped with a robotic Elekta HexaPOD evo RT System couch.

To ensure that the observed deviations were due to residual patient motion in the thermoplastic mask rather than geometric inaccuracies of the linac setup, we compared patient data with results from a dedicated phantom study. For the phantom study, a head phantom was positioned to simulate a patient treatment, under two different scenarios: with and without an additional load on the treatment couch to mimic patient‐like weight.

## 2 MATERIALS AND METHODS

The ETD system was used to detect the initial intrafraction motion occurring after couch rotations during non‐coplanar stereotactic treatment delivery. This system consists of a combination of a stereoscopic X‐ray imaging system with a surface‐guided radiotherapy (SGRT) system and additional thermal information.^42^

The X‐ray component, which is the most relevant system for this study, acquires images using two floor‐mounted X‐ray tubes projecting obliquely onto two flat panel detectors on the ceiling. The stereoscopic kilovolt (kV) X‐rays are used to verify the bony anatomy, by rigidly matching and registering the X‐ray images to the digitally reconstructed radiographs (DRRs), from the treatment plan, resulting in a 6DoF correction shift.^37^ The resulting shifts, if larger than the specified tolerances, must be sent from ETD to the robotic Elekta HexaPOD evo RT System couch, via the iGuide software Version 2.2.2 (Elekta AB, Stockholm, Sweden), to move the patient to the planned treatment position. The patient is considered to be accurately positioned if the remaining deviation between the actual and the planned position is below the defined tolerances. An optical surface reference is created after the acquisition of each pair of X‐rays and it is then updated with every stereoscopic X‐rays.^43^

The SGRT component is a hybrid optical surface and thermal imaging device, containing a blue light projector, two stereoscopic cameras and an integrated thermal camera. The system acquires real‐time surface data and integrates an additional thermal signature, thereby increasing the registration accuracy.^42^, ^43^ This system allows a permanent and continuous surveillance of the patient´s surface throughout the entire treatment delivery.

Considering that only closed double‐layered thermoplastic stereotactic mask systems were utilized in this study, insufficient patient surface was visible for surface tracking. Therefore, only X‐ray data were used to analyze intrafraction deviations.^30^, ^44^

Two studies were conducted: a patient study and a phantom study. The ETD system, version 1.1.1 (Brainlab SE, Munich, Germany) was used for initial patient positioning and for intrafraction monitoring throughout each fraction, by acquiring stereoscopic X‐rays every 90° of gantry rotation and after each couch rotation. The stereoscopic X‐ray images enable the detection and immediate correction of any intrafraction motion exceeding predefined limits. Similarly, in the phantom study, stereoscopic X‐rays were acquired after each new couch rotation to assess the deviations caused by the couch rotation.

### 2.1 Patient study

To assess intrafraction deviations in non‐coplanar intracranial radiosurgery treatments, anonymized intrafraction X‐ray data were collected from 50 patients treated for brain metastases between August 2021 and September 2022 at the Department of Radiation Oncology, University Hospital, LMU Munich. The selected patients were recruited in a prospective study (Ethics protocol 20–0664). Written informed consent was obtained from all participants.

### 2.2 Clinical workflow

Patients were fixed with an individually moulded double‐layered thermoplastic stereotactic face mask (from Brainlab SE, Munich, Germany or IT‐V, Innsbruck, Austria) during planning CT and treatment. The planning CT was performed at a Toshiba Aquilion LB (Canon Medical Systems, Japan) CT scanner in helical mode with a slice resolution of 1 mm and an in‐plane resolution of 1.074 mm x 1.074 mm.

For treatment planning GTVs were delineated by an experienced radiation oncologist, according to the institutional protocol, and a margin of 1 mm was applied to create the PTV.

For all patients included in this study, plans were generated using a 6MV photon beam and all of them included non‐coplanar couch angles. For patients with multiple lesions the treatment planning system (TPS) Multiple Brain Mets SRS Versions 2.0.0 and 3.0.0 (Brainlab SE, Munich, Germany) were utilized to create dynamic conformal arc therapy (DCAT) plans. The treatment isocenter was automatically defined by the TPS to the geometrical average of the centre‐of‐mass of all lesions. According to our institutional protocol, all lesions must be located within 70 mm distance to the isocenter, otherwise two isocenters are used.

Patients with single lesions received a volumetric arc therapy (VMAT) plan, generated by the TPS Monaco Version 5.51.10 (Elekta AB, Stockholm, Sweden).

Treatments were delivered on an Elekta Versa HD linac with an Agility multileaf collimator (160 leaves, leaf width 5 mm; Elekta AB, Stockholm, Sweden) equipped with an Elekta HexaPOD evo RT robotic couch capable of 6DoF corrections and providing an overall mean positioning accuracy of < 0.5 mm and < 0.025° for translational and rotational error, respectively.^45^ For initial patient positioning and monitoring during treatment ETD system, version 1.1.1 (Brainlab SE, Munich, Germany) was used.

Initial patient positioning at 0° couch angle was performed using ETD stereoscopic kV X‐ray imaging of the cranial bony anatomy and registered to DRRs. The resulting 6DoF X‐ray‐based correction shift (3 translations and 3 rotations) was sent and applied via the iGuide software to the robotic HexaPOD couch, followed by a second pair of X‐ray images to verify the corrected position. The communication between the ETD system and the linac is made through the Elekta Response gating control interface (Elekta AB, Stockholm, Sweden), responsible for enabling beam‐on once all the requirements are fulfilled, that is, once all deviations are below the predefined tolerances. During treatment delivery (beam‐on), for patient position monitoring purposes, stereoscopic X‐ray images were acquired at cardinal gantry angles. If deviations between planned and actual position, detected automatically through the registration of X‐ray images and DRRs, remained within tolerance, treatment continued without correction. Whenever deviations exceeding predefined tolerance levels were detected, beam delivery was interrupted, the fusion of the X‐ray images and the corresponding DRRs was analyzed by a trained physician or radiation therapist, the calculated 6DoF correction shift was then sent, from the control room, to the robotic couch via the iGuide software and the patient was re‐positioned accordingly. Stereoscopic X‐ray images were also and must be always acquired after every couch rotation before the treatment was resumed, to ensure accurate patient positioning at each non‐coplanar couch angle. The predefined tolerance levels of 0.5 mm for translations and 0.5° for rotations were chosen for all SRS and FSRT treatments.

Before each stereotactic treatment a WL test was performed to verify the alignment of the ETD's imaging isocenter and the linac's radiation isocenter, for which the recommended accuracy is 1 mm for SRS treatments.^46^, ^47^, ^48^ This test was conducted with a cranial anthropomorphic head phantom (Brainlab SE, Munich, Germany), with the intention of resembling the traditional workflow of IGRT‐based treatments, well‐known as the hidden target test. This phantom with a 5 mm‐diameter inserted ball bearing, was fixed to the treatment couch with a cranial 4Pi stereotactic immobilization system (Brainlab SE, Munich, Germany) (see Figure 2a and b), and positioned with ETD. A treatment plan with four square fields (2 × 2 cm^2^) centered at isocenter (defined at the centre of the ball bearing), was delivered with gantry at cardinal angles. If calibration of the ETD imaging isocenter was required, a WL pointer phantom (Brainlab SE, Munich, Germany), in the form of a 5 mm‐diameter tungsten ball embedded in a plastic structure, was attached to the treatment couch and used as a target for calibration. Following ETD's calibration, a new hidden target test was performed. The results of hidden target tests, performed throughout one year, were included and are shown in the Phantom Study section.

Furthermore, image quality assurance is automatically performed by frequent measurements with the anthropomorphic phantom, where the consistency of the results is evaluated and compared.

### 2.3 Data analysis

For the present study, information regarding all 6DoF X‐ray‐based correction shifts was retrieved from the patient positioning reports, generated in a PDF‐format after every treatment session. For the analysis, the first uncorrected deviations recorded after each couch rotation was considered.

To check for correlations between magnitude of deviations and treatment duration, monitor units (MU) and number of couch angles were used as surrogates, since actual treatment times could not be retrieved retrospectively.

Intrafraction X‐ray data of 26 patients treated with single‐fraction SRS were collected, being analyzed 40 treatments in total (11 patients received more than one SRS treatment). Intracranial lesions (1–15 lesions, mean = 2.4, median = 1), were treated with a median dose of 20 Gy prescribed to the 80% isodose. A median of 5 couch angles per plan, ranging from 3 to 8, was used for treatment delivery. In total, 168 stereoscopic X‐rays were acquired and evaluated to assess the first measured positioning deviation after each couch rotation.

Regarding the FSRT treatments (median number of fractions per treatment of 5), 24 patients (with one or two lesions) were included and received a median applied dose of 25 Gy prescribed to the 80% isodose. A median of 3 couch angles per plan, ranging from 2 to 4, was used to deliver the treatments. Overall, 128 fractions with 278 stereoscopic X‐ray images, performed after a couch rotation, were evaluated. Similar to the SRS treatments, the first measured setup deviation after each couch rotation was assessed.

For both SRS and FSRT treatments and all sessions, a deviation vector was calculated similarly to the approach described by D. Reitz et al.,^29^ in order to quantify the absolute translational deviation:

(1) $$d=\sqrt{x^{2}+y^{2}+z^{2}}=\sqrt{lateral^{2}+longitudinal^{2}+vertical^{2}}\;$$

**FIGURE 1:**
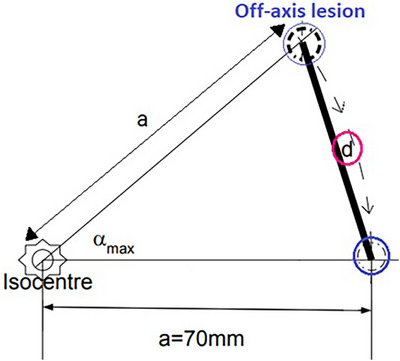
A schematic drawing of the geometry of a maximum rotational deviation of a lesion, *α_max_*, located at a maximum allowed distance of 70 mm from the isocenter, resulting in a translational deviation, *d*.

Additionally, we investigated the maximum rotational deviation (the largest observed rotation among all three rotation corrections: pitch, roll and yaw) across all treatments from the 50 selected patients, α_max_. As cranial lesions are typically located within 70 mm from isocenter, we aimed at illustrating the potential impact of rotational deviations for a hypothetical lesion located at a distance *a* = 70 mm from the isocenter, and calculated the resulting translational displacement, *d*, as shown in Figure 1.

### 2.4 Phantom study

**FIGURE 2:**
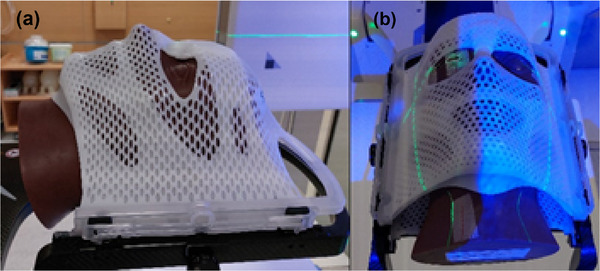
a and b: Image of the anthropomorphic head phantom, immobilized to the treatment couch with a thermoplastic cranial 4Pi stereotactic face mask.

To distinguish displacements caused by couch rotation from real patient motion, another study was performed using an anthropomorphic head phantom (Brainlab SE, Munich, Germany). The phantom was fixed to the treatment couch with a thermoplastic cranial 4Pi stereotactic immobilization face mask (double‐layered mask, Brainlab SE, Munich, Germany), shown in Figure 2a and b.

**FIGURE 3:**
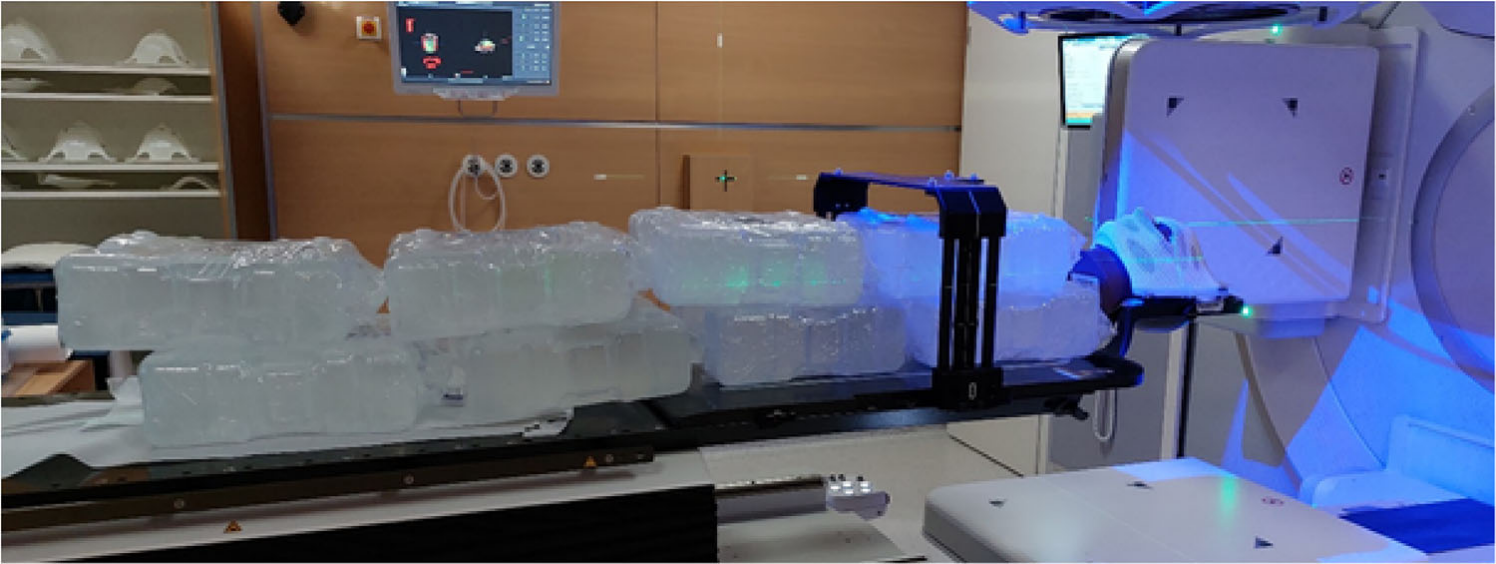
Experiment with 80 kg weight (water containers) evenly distributed on the treatment couch to simulate a patient, together with an anthropomorphic cranial verification phantom, immobilized with a cranial 4Pi stereotactic mask.

Stereoscopic X‐rays were acquired for 7 equidistant couch rotations (0°, 30°, 60°, 90°, 270°, 300° and 330°) under two conditions: without additional weight on the couch and with 80 kg weight evenly distributed, to simulate a patient (see Figure 3). Each scenario was performed 25 times (175 measurements each, 350 measurements in total).

The ETD radiation isocenter was calibrated before carrying out the phantom study. For the calibration, a WL pointer phantom (Brainlab SE, Munich, Germany) was attached to the treatment couch and used as a target, as already mentioned.

### 2.5 Statistical analysis

The IBM SPSS Statistics Version 29.0 software (IBM Corporation, Armonk, New York) was used to perform Mann‐Whitney U tests to investigate differences of the positioning deviations measured by stereoscopic X‐ray imaging between SRS and FSRT treatments, as well as between an unloaded treatment couch and a couch loaded with 80 kg weight. Differences were considered statistically significant for *p* value ≤0.05. Data processing was performed using Microsoft Excel 365 (Microsoft Corp.).

## 3 RESULTS

### 3.1 Patient study

For the 40 single‐fraction SRS treatments (26 patients), a total of 168 stereoscopic X‐ray images were analyzed and compared to the DRRs from the treatment plan. Table 1 summarizes the number of deviations, translations and rotations detected relative to the treatment couch position, exceeding the clinically applied repositioning tolerances of ≥ 0.5 mm/0.5°. Furthermore, the number and percentage of deviations for the theoretical scenario of applying a higher tolerance level of 1 mm/1° are also reported.

**TABLE 1:** Number and percentage of deviations beyond the clinically defined tolerances (0.5 mm/0.5°) and a theoretical scenario of higher tolerance levels of 1 mm/1° from 40 single‐fraction SRS treatments, based on 168 stereoscopic X‐rays acquired after couch rotations.

Translational and rotational deviations							
	Lateral	Longitudinal	Vertical	Pitch	Roll	Yaw	Any
*N* of stereoscopic X‐rays acquired	168	168	168	168	168	168	168
*N* deviations ≥ 0.5 (mm/°)	**28 (16.7%)**	18 (10.7%)	3 (1.8%)	2 (1.2%)	3 (1.8%)	**41 (24.4%)**	**75 (44.6%)**
*N* deviations ≥ 1 (mm/°)	2 (1.2%)	2 (1.2%)	2 (1.2%)	0 (0%)	0 (0%)	3 (1.8%)	5 (3%)

Patients required repositioning 75 times (45%) after couch rotations, as the clinically adopted tolerance threshold (deviations ≥ 0.5 mm/0.5°) was exceeded at least in one axis. Deviations involving 2 or more DoF occurred 15 times, representing 9% of the total number of verification images. Notably, 97% of all deviations were below the wider threshold of 1 mm/1°.

**FIGURE 4:**
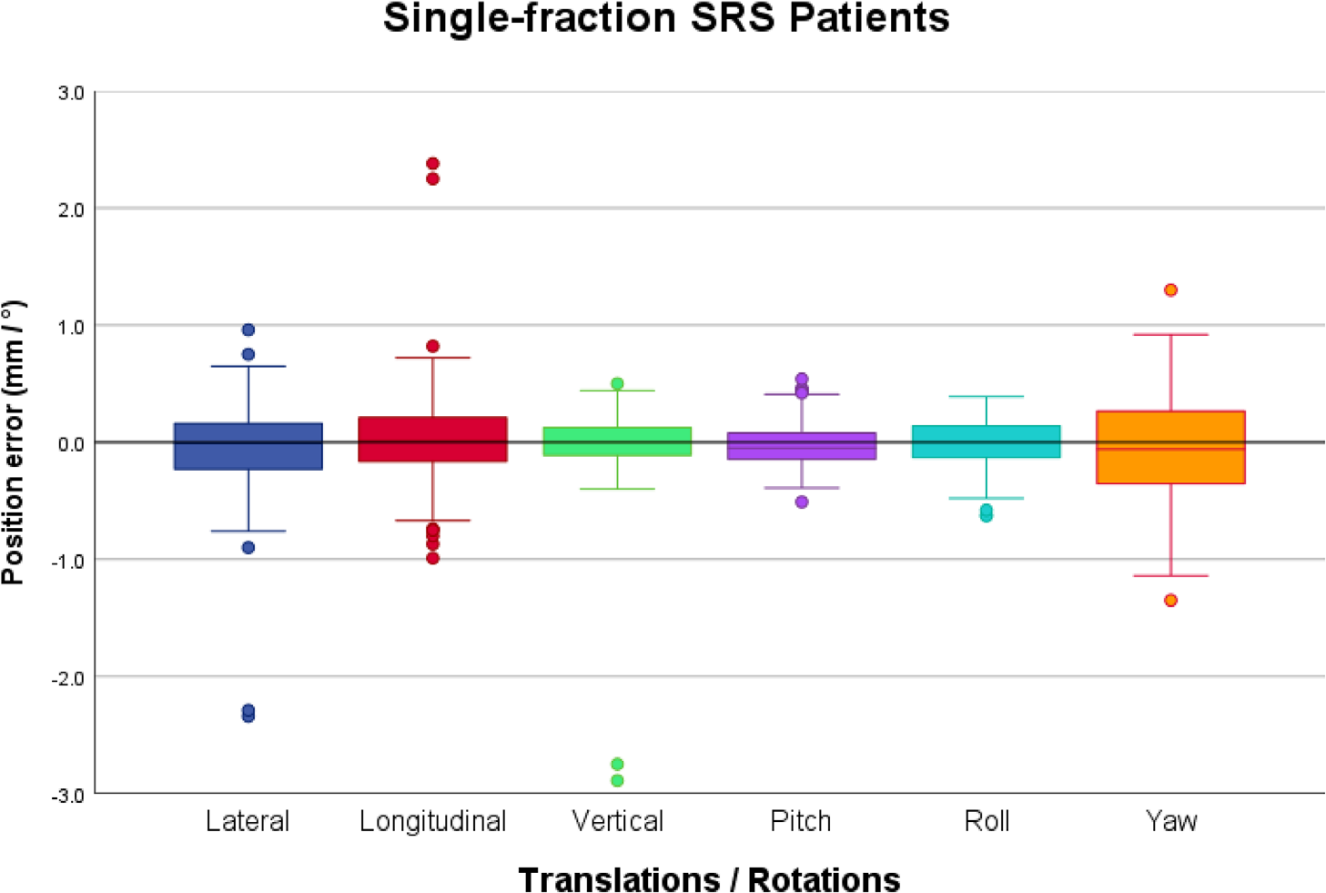
Boxplots of the distribution of the intrafraction deviations after each couch rotation during 40 SRS treatments (168 stereoscopic X‐rays). The boxplots indicate the spread of the central 50% of the data, denominated as IQR. The median, the 25^th^ (Q_1_) and the 75^th^ (Q_3_) percentiles are also shown. The upper and lower whiskers represent data outside the IQR but inside the range defined by 1.5 × IQR. Outliers are defined as values outside the whiskers’ range.

Figure 4 gives an overview of the intrafraction deviations after couch rotation, in all 6 DoF:

Although the largest absolute translational deviation was observed in the vertical direction, deviations exceeding the tolerance level were most frequent in the lateral (17%) and yaw (24%) directions. Maximum translational deviations reached 2.3 mm, 2.4 mm and 2.9 mm in the lateral, longitudinal and vertical direction, respectively. However, the 2 mm threshold was exceeded only for 2 fractions and for both fractions in all 3 translational directions simultaneously (in Figure 4 the 2 fractions with magnitude > 2 mm are represented by the outliers in all 3 translational directions). Excluding these two cases, the maximum magnitude of deviations was less than 1 mm in all translational directions. Maximum rotational deviations were 0.5°, 0.6° and 1.4° for pitch, roll and yaw, respectively.

The spread of the central 50% of the data (between the 25^th^ and the 75^th^ percentile) was always below 0.25 mm and not higher than 0.35° regarding the absolute translational and rotational deviations, respectively.

The distribution of the deviations was centred around the origin, with maximum median deviations reaching 0.02 mm in the lateral direction and 0.06° observed in the yaw axis.

Regarding treatment duration, the SRS treatment plans had a median of 3834 MU, ranging from 2954 to 19419 MU.

For the 128 FSRT treatment fractions (24 patients), Table 2 provides an overview of the 278 stereoscopic X‐ray images analyzed, along with the number and percentage of deviations exceeding the defined tolerances for repositioning the patient.

**TABLE 2:** Number and percentage of deviations beyond the clinically defined tolerances (0.5 mm/0.5°) and a theoretical scenario of higher tolerance levels of 1 mm/1° from 128 FSRT fractions, based on 278 stereoscopic X‐rays acquired after couch rotations.

Translational and rotational deviations							
	Lateral	Longitudinal	Vertical	Pitch	Roll	Yaw	Any
*N* of stereoscopic X‐rays acquired	278	278	278	278	278	278	278
*N* deviations ≥ 0.5 (mm/°)	**62 (22.3%)**	59 (21.2%)	6 (2.2%)	6 (2.2%)	3 (1.1%)	**68 (24.5%)**	**151 (54.3%)**
*N* deviations ≥ 1 (mm/°)	1 (0.4%)	2 (0.7%)	0 (0%)	0 (0%)	0 (0%)	9 (3.2%)	12 (4.6%)

Patient repositioning was required 151 times after couch rotations (deviations ≥ 0.5 mm/0.5°), corresponding to 54% of all verifications. Tolerances were exceeded in 2 or more DoF on 43 occasions (15%).

**FIGURE 5:**
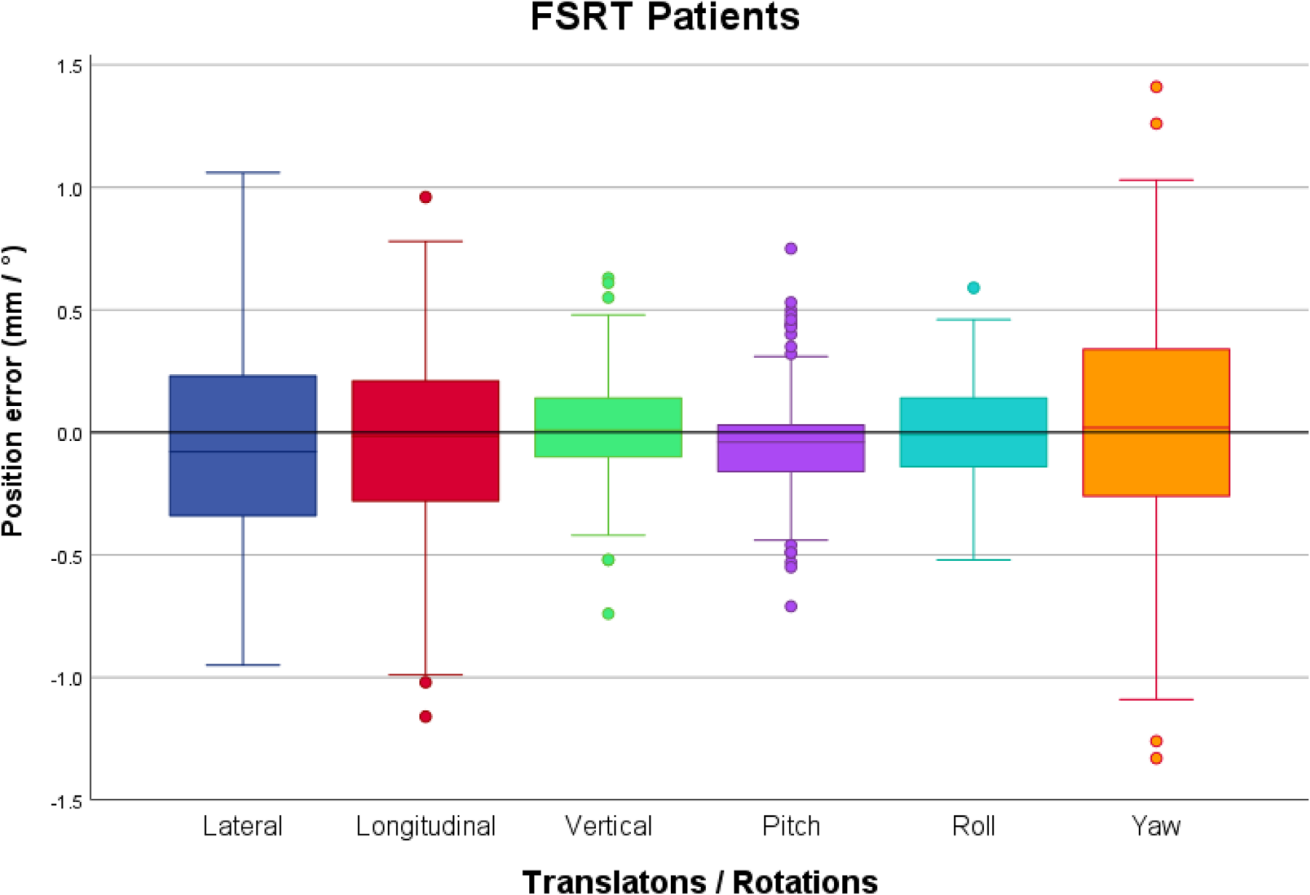
Boxplots of the distribution of intrafraction deviations after each couch rotation during 128 FSRT fractions (278 stereoscopic X‐rays). The boxplots indicate the spread of the central 50% of the data, denominated as IQR. The median, the 25^th^ (Q_1_) and the 75^th^ (Q_3_) percentiles are also shown. The upper and lower whiskers represent data outside the IQR but inside the range defined by 1.5 × IQR. Outliers are defined as values outside the whiskers’ range.

In case of a theoretical higher tolerance level of ≥ 1 mm/1°, repositioning would have been necessary in 4% of cases. The distribution of the observed deviations in all 6DoF after each couch rotation is shown in Figure 5.

Most translational deviations out of tolerance occurred in the lateral (22%) and longitudinal (21%) axis. For rotational deviations, the yaw axis was most frequently out of tolerance (25%). Maximum translational deviations were 1.1 mm, 1.2 mm and 0.7 mm in the lateral, longitudinal and vertical direction, respectively. Regarding rotational deviations, the maximum observed errors were 0.8°, 0.6° and 1.4° for pitch, roll and yaw axis, respectively. The interquartile range (IQR), the central 50% of the data between Q1 and Q3, indicates the likely range of variation, and for FSRT treatments the absolute translational and rotational deviations between the 25^th^ and the 75^th^ percentile was below 0.35 mm and 0.35°, respectively.

Similar to the single‐fraction treatments, the distribution of the deviations was bi‐directional for all translational and rotational axis, with maximal median deviations reaching 0.08 mm in the lateral direction and 0.04° observed in the pitch axis. Concerning the duration of treatment, FSRT treatment plans were significantly shorter with a median of 1243 MU, ranging from 564 to 2607 MU.

For both SRS and FSRT treatments and all fractions, a deviation vector was calculated according to equation (1), excluding the two outliers (see Table 3):

**TABLE 3:** The deviation vector calculated according to equation (1), quantifying the absolute translational deviation, for both SRS and FSRT treatments.

	SRS treatments	FSRT treatments
Mean translation (mm):	0.4 (SD 0.2 mm)	0.5 (SD 0.3 mm)
Median translation (mm):	0.4	0.5
Maximum translation (mm):	1.1	1.4

Regarding rotational deviations, the largest rotational deviation detected was *α_max _= 1.4°* in the yaw direction for both SRS and FSRT treatments. For a hypothetical lesion located at a maximum distance of *a = 70 mm* of the isocenter, this corresponds to a calculated translational deviation *d = 1.7 mm* (see Figure 1).

A Mann‐Whitney U test was performed to investigate whether the differences reported by the X‐ray imaging positioning values for both SRS and FSRT treatments were statistically significant. The test showed that there was no significant difference in all translations as well as in rotations (*p* > 0.05), except for yaw which presented *p *= 0.042, when comparing an SRS treatment to an FSRT treatment.

### 3.2 Phantom study

Hidden target tests were performed on the same day as the stereotactic treatments, representing the residual differences between the ETD's imaging isocenter and the radiation field isocenter. In table 4, the deviations obtained from our hidden target tests, performed throughout one year, are shown:

**TABLE 4:** Deviations resulting from hidden target tests, performed on the same day as the stereotactic treatments.

Translational deviations				
	Lateral	Longitudinal	Vertical	Vector length
Mean (mm)	0.02	−0.03	−0.14	0.30
Standard Deviation (mm)	0.20	0.17	0.17	0.16

Moreover, in the phantom study, 175 displacements were measured in two different scenarios: with and without additional weight on the treatment couch.

**FIGURE 6:**
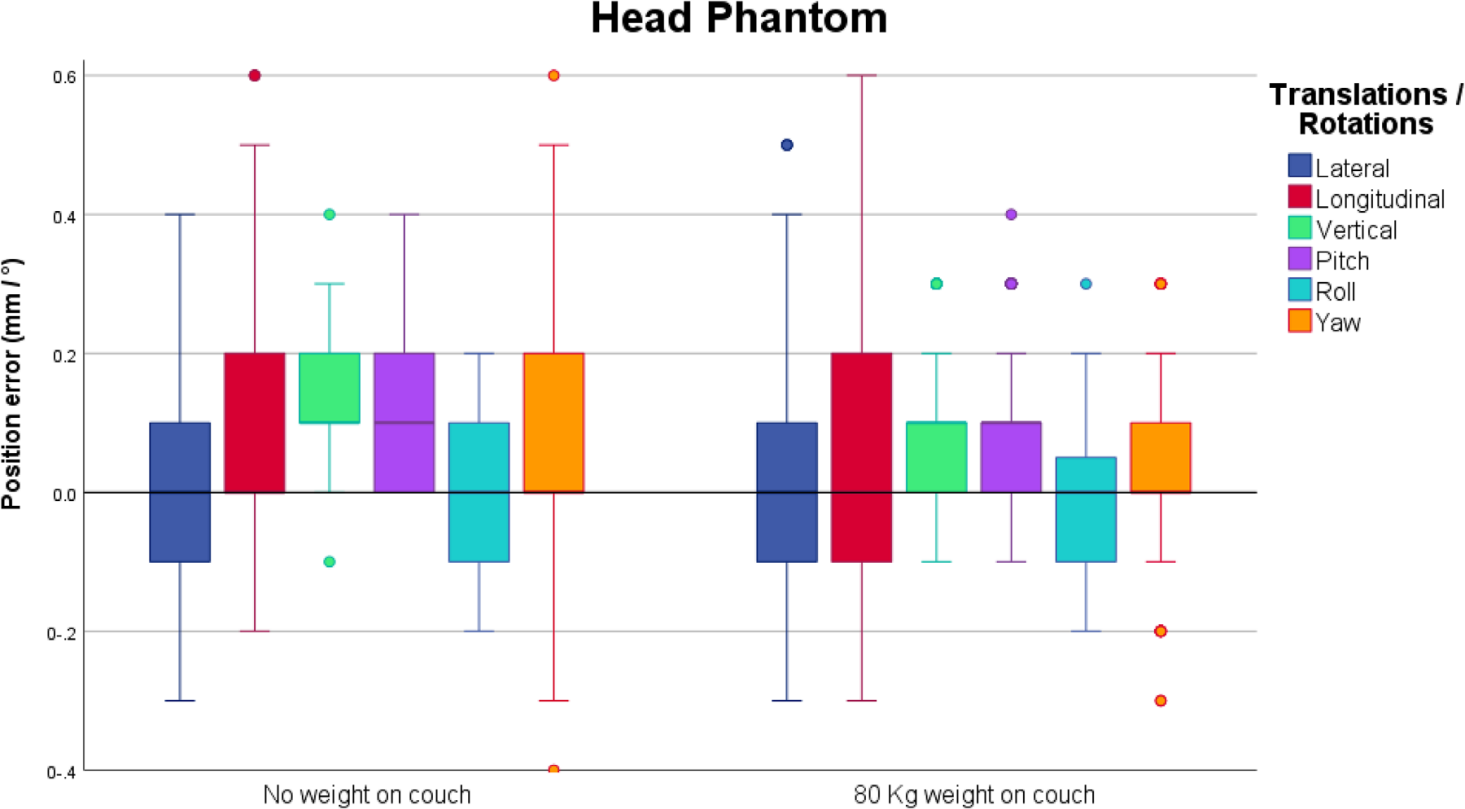
Boxplots of the distribution of the intrafraction deviations acquired and recorded after each of 7 equidistant couch rotations (0°, 30°, 60°, 90°, 270°, 300° and 330°), through stereoscopic X‐rays, for both scenarios. The boxplots indicate the spread of the central 50% of the data, denominated as IQR. The median, the 25^th^ (Q_1_) and the 75^th^ (Q_3_) percentiles are also shown. The upper and lower whiskers represent data outside the IQR but inside the range defined by 1.5 × IQR. Outliers are defined as values outside the whiskers’ range.

Figure 6 represents the spread of the intrafraction deviations in all 6DoF, observed throughout 350 measurements.

Comparably to the patient study, the distribution of the deviations was characterized by median deviations of 0 or close to 0 mm, and the largest median deviation observed was 0.1 mm.

In both scenarios there were some deviations reaching 0.5 mm or even slightly exceeding the tolerance by 0.1 mm. Regarding the measurements performed without weight on the treatment couch, 5.7% of all measurements reached at least a deviation of 0.5 mm in the longitudinal direction, and 4% in the yaw direction. Similar results were obtained when simulating a patient on the couch, with 80 kg weight placed on the treatment couch and deviations reaching at least 0.5 mm were seen on the lateral (1.7%) and longitudinal (6.3%) directions.

Slightly smaller deviations in the vertical, pitch and yaw directions were recorded, when the couch was under weight.

A Mann‐Whitney U test was performed to investigate whether the differences reported by the X‐ray imaging positioning values for both situations, without additional weight and with 80 kg on the couch, were statistically significant or not. The test showed that the results were significantly different for the vertical direction (*p* < 0.001) as well as for pitch (*p* = 0.016) and yaw (*p* = 0.035), even though the median values were the same for both cases. Furthermore, when analysing the likely range of variation for both situations by comparing the IQR, a maximal difference of 0.1 mm and 0.1° for each direction was observed. The same observation can be made regarding maximal displacement values, except for yaw, where a larger difference of 0.3 mm was seen.

## 4 DISCUSSION

To investigate the impact of couch rotations on patient positioning in non‐coplanar stereotactic treatments, two different studies were performed: a patient study including 50 patients (26 treated with single‐fraction SRS and 24 with FSRT), as well as a phantom study. The latter was carried out to distinguish true patient motion from deviations caused by couch rotations or system‐related effects.

While many systems do not provide IGRT at couch angles other than 0°, this is, to the best of our knowledge, the first study specifically addressing patient motion after couch rotations in stereotactic cranial treatments when combining an Elekta Versa HD linac, an Elekta HexaPOD evo RT robotic couch and the ETD system used during the whole fraction in different stages: for a correct and accurate final patient position, for position monitoring and to enable patient reposition throughout the entire treatment during beam‐on and beam‐off time. Our aim was to assess the magnitude of these deviations and to determine whether their correction was clinically relevant.

In order to quantify deviations arising exclusively from couch rotations and patient setup itself, a dedicated phantom study was conducted. In total, 350 measurements were analyzed to assess the magnitude and variability of these deviations under controlled conditions. When a patient‐like weight was applied to the treatment couch to simulate a real treatment situation, the variability of the measurements (IQR) was slightly narrower or equal compared to the unloaded couch, except in the longitudinal direction. Furthermore, the comparison between the two couch load conditions showed statistically significant differences in the vertical, pitch and yaw directions. However, when comparing the IQR and the absolute maximal deviations for both weight loads, the differences < 0.1 mm in all but the yaw direction. This indicates minimal variance between the two weight conditions and demonstrates that the treatment couch maintains comparable accuracy under different loading. For both conditions, the IQR did not exceed 0.2 mm and 0.2°. When simulating a patient treatment, maximum deviations of up to 0.6 mm and 0.4° were observed. A similar study recorded larger translational deviations at different couch angles under varying weight loads.^23^ Although all translational couch position deviations were within 1 mm, they were generally greater than in our study, except in the vertical direction, which was least affected. These findings could be correlated to the treatment couch itself, its stability and alignment that may differ from couch to couch.

Concerning our patient study, we report clinical positioning deviations after couch rotations recorded from 446 intrafraction stereoscopic X‐ray images (168 pairs of X‐rays acquired during 40 single‐fraction SRS treatments and 278 pairs of X‐rays during 128 FSRT fractions). For both SRS and FSRT treatments, the distribution of deviations was bi‐directional across all translational and rotational axes and centred around the origin, and the median of all deviations did not exceed 0.09 mm and 0.06°, as expected.

The IQR of the data was also very similar for both treatment types and did not exceed 0.35 mm or 0.35° for translational and rotational deviations, respectively. Although not statistically significant, when observing the boxplots of the deviations for both treatment types, a slightly higher absolute maximum translational deviations were observed for SRS treatments (2.3 mm, 2.4 mm and 2.9 mm in the lateral, longitudinal and vertical directions, respectively), whereas rotational deviations were comparable between SRS and FSRT. These findings might be correlated with the higher number of non‐coplanar couch angles (median of 5 couch angles for SRS whilst FSRT had a median of 3 couch angles), a larger number of lesions treated, the higher delivered dose, the higher number of MU, which is associated with a higher complexity of the plan as well as a longer treatment delivery time for SRS treatments. Nevertheless, it is also important to note that translational deviations greater than 2 mm occurred only in two measurements across the 168 stereoscopic X‐rays, corresponding to 1.2% of all recorded deviations. These were, therefore, considered outliers and not representative of the deviations most commonly observed. Excluding these two cases, the maximum deviations were 1 mm in the lateral and longitudinal directions and 0.5 mm in the vertical direction. When comparing our results with other studies in the literature, such as Badakhshi et al.,^19^ which investigated patients undergoing single‐fraction SRS on a Novalis linac equipped with a Varian robotic couch of similar positioning accuracy^49^ and several non‐coplanar arcs, relevant similarities were observed. They reported mean intrafraction translational errors of 0.8 mm, 0.8 mm and 0.7 mm for the lateral, longitudinal and vertical directions, respectively. Moreover, the frequency of the translational deviations exceeding 1, 2 and 3 mm were respectively: 12%, 3% and 1% in all three directions.^19^ Overall, our positioning deviations were slightly smaller, but nevertheless comparable. One possible explanation for our better results could be the use of a less tight thermoplastic mask in their study, as the vendor and mask type were not specified. In a more recent study, Camels et al. applied a similar approach using a Varian TrueBeam linac and achieved equivalent results.^50^ Moreover, Tanaka et al. investigated translational deviations in stereotactic treatments with non‐coplanar couch angles, using a Novalis‐Tx linac and an ExacTrac system version 6.0.6 and reported values consistently below 2 mm.^23^

When evaluating how frequent deviations exceeded the clinically applied tolerance levels (≥ 0.5 mm/0.5°), we found that SRS patients required repositioning after 45% of couch rotations, whereas for FSRT this value was slightly higher at 54%. Agazaryan et al. reported similar findings, noting that 51.4% of patients exceeded the determined tolerance level of 0.5 mm in the translational direction.^39^ However, and differently from our clinical workflow, this percentage did not include rotational deviations, as robotic rotational corrections were not applied.^39^

Additionally, the majority of out‐of‐tolerance deviations was detected in the lateral direction for both treatment types: in 17% of the stereoscopic images performed during SRS treatments and 22% for FSRT. These percentages are quite similar and their predominance in the lateral direction may be attributed to the couch rotation, which can induce a movement of the patient along the same axis. For rotational errors, both treatment types showed similar patterns, with most deviations occurring in the yaw direction (24% in SRS and 25% in FSRT treatments). The maximum rotational deviation observed was 1.4°, while the median deviation remained below 0.1° for both treatment modalities.

In the hypothetical scenario where the clinical tolerance threshold was set to 1 mm, the frequency of a necessary repositioning for SRS and FSRT treatments would decrease to 3% and 4%, respectively. Accordingly, in 97% and 96% of all couch rotations, patient deviations remained within 1 mm and 1°, which is consistent with the findings of M. De Ornelas et al.,^6^ However, these results contrast with the outcomes reported by Badakhshi et al., who found 12% of misplacements exceeding 1 mm.^19^ Another study reported the following positional accuracy within 1 mm after rotating the treatment couch: 77.86%, 72.26% and 98.47% for the lateral, longitudinal and vertical directions, respectively. In contrast, in our study each of the 3 translational deviations remained < 1 mm in at least 99% of the cases. The differences observed between our results and the results presented in similar studies may result from our more comprehensive verification strategy: patients’ positioning was not only verified and corrected after each couch rotation but also continuously monitored using the surface/thermal camera system. In addition, patients were frequently imaged during beam‐on time (4 pairs of X‐rays were acquired across 360° gantry rotation) with corrections applied whenever deviations exceeded predefined tolerances. This repeated monitoring and possible deviation correction throughout the entire treatment might perhaps result in a further decrease of residual deviations throughout treatment, namely before each couch rotation. Additionally, our phantom study indicated that positional deviations attributable solely to couch rotations were minimal. Another factor contributing to the relatively small correction deviations after couch rotations may potentially also be explained by the type of immobilization masks used for patient fixation. As demonstrated in the literature, patient immobilization provided by stereoscopic double‐layered thermoplastic masks has proved to allow very little patient movement during treatment (median below 0.3 mm), when compared to open face masks, used in non‐SRS treatments.^20^ The study showed that treatments in stereotactic mask systems took longer but nevertheless presented significantly smaller deviations when compared to treatments with open‐face mask systems, and the median magnitude of the deviation vector was 0.3 mm in the non‐coplanar setting for stereotactic mask systems, and 0.4 mm for the open mask systems.^20^ Regarding treatment time for cranial lesions, frequent ETD‐based monitoring does not necessarily prolong treatment time, since the stereoscopic X‐ray images are acquired simultaneously with treatment delivery (during beam‐on). The registration between X‐ray images and DRRs is performed automatically and as long as the deviation between actual and planned patient position does not exceed the predefined limits (on the ETD system), the treatment is continued. It is important, however, to consider that if the detected deviations exceed the specified tolerances, the treatment is interrupted. The treatment is then resumed once the patient is correctly repositioned. Furthermore, after each couch rotation, another pair of X‐rays is acquired and from our results, repositioning was required in 45% and in 54% of all verifications, for SRS and FSRT treatments respectively. By our experience, around 10% of the total treatment time is needed for patient repositioning.

Regarding the 3D‐vector magnitude, it reached a median value of 0.4 mm and a mean value of 0.4 mm (SD 0.2 mm) for SRS treatments. For FSRT treatments the results were slightly higher, however comparable, achieving the following median and mean: 0.6 mm and 0.5 mm (SD 0.3 mm). Agazaryan et al. also reported a mean 3D deviation of 0.64 mm (SD 0.12),^39^ while Badakhshi et al. observed a higher mean vector magnitude of 1 mm (SD 0.9 mm).^19^

Although less common, some absolute deviations reached 2 mm. Even if these outliers were excluded, the maximum deviation still exceeded 1 mm and 1°. Considering rotational deviations, a maximum rotation of 1.4° was recorded in the yaw direction. This could induce a translational shift of 1.7 mm, if a lesion would be located at the maximum lesion‐to‐isocenter distance allowed at our institution (70 mm). In such a scenario, the applied 1 mm PTV margin would not be sufficient to fully account for all uncertainties present during stereotactic treatments. Our findings go in line with Tanaka et al., as they reported maximum deviations exceeding the adopted PTV margins used in stereotactic treatments.^23^ Moreover, our results demonstrate that even in spite of a frequent intrafraction motion monitoring and repositioning in cranial stereotactic treatments while beam‐on, and despite the fact that patients were immobilized with stereotactic closed double‐layered masks, patient repositioning when non‐coplanar beams were employed was still required in nearly half of all verifications, which we consider therefore essential to ensure a safe, accurate and precise dose delivery. Performing frequently the WL test, as well as the imaging isocenter calibration, to ensure the alignment between both imaging and radiation isocenters is pivotal, to minimize the geometrical uncertainties, and therefore increase the probability of the CTV to receive the planned dose. By executing regularly this test, our results obtained from hidden target tests performed throughout one year showed a mean and standard deviation of 0.3 ± 0.16 mm.

Furthermore, implementing tolerance levels of ≥ 0.5 mm/0.5° for X‐rays that are tighter than the PTV margin is necessary to adequately account for any residual uncertainties.

In our study all patients were positioned with thermoplastic closed double‐layered masks due to their higher immobilization capability.^20^ However, for an optical system to be able to correctly and accurately position a patient, to provide continuous surveillance and to be capable of detecting patient positioning deviations, sufficient patient surface information is crucial. For cranial treatments, this necessity is translated by immobilising patients with open‐face masks, so that enough surface information is available. A study by D. Reitz et al.^20^ reported significant larger deviations for open‐face masks, demonstrating the possibility for more patient movement. Moreover, the use of SGRT systems has shown to be prone to other errors in the treatment of cranial lesions, such as facial expressions, which may generate false positive corrections and therefore impact the delivery of the treatment.^51^, ^52^ Taking these uncertainties into account, larger PTV margins would be required if an SGRT‐only workflow was used, especially solely. However, increased safety margins are also correlated to an undesirable increase in the risk of radiation necrosis.^52^

## 5 CONCLUSIONS

This study is the first to specifically assess patient motion after couch rotations in cranial non‐coplanar stereotactic treatments on an Elekta Versa HD using stereoscopic X‐ray imaging for IGRT. While phantom experiments confirmed that couch rotations themselves introduce only negligible deviations (0.6 mm and 0.6°), patient data revealed that clinically relevant shifts do occur, representing true patient motion despite rigid frameless immobilization. Repositioning was required in nearly half of all couch rotations when applying the clinical tolerance of ≥ 0.5 mm/0.5°, with most deviations occurring laterally and in yaw direction. Although over 96% of all deviations remained within 1 mm/1°, occasional outliers exceeded 2 mm or 1.4°. These findings demonstrate that correcting such patient motion is clinically relevant to ensure a highly precise and safe stereotactic treatment.

## AUTHOR CONTRIBUTIONS

Vanessa da Silva Mendes, Stefanie Corradini and Michael Reiner drafted the manuscript. Vanessa da Silva Mendes and Michael Reiner performed the measurements and analysis of the data, performed the statistical analysis. Stefanie Corradini, Sylvia Garny, Lili Huang, Stephan Schönecker, Christian Trapp and Frederik Fuchs assisted in clinical data collection, supervised the analysis, reviewed the manuscript and helped finalize it. Christopher Kurz, Guillaume Landry helped design the study, supervised the analysis, reviewed the manuscript and helped finalize it. Stefanie Corradini, Michael Reiner and Claus Belka conceived the study, helped design the study, supervised the analysis, reviewed the manuscript and helped finalize it. All authors read and approved the final manuscript.

## CONFLICT OF INTEREST STATEMENT

The authors declare the following financial interests/personal relationships which may be considered as potential competing interests: the Department of Radiation Oncology of the LMU University Hospital, LMU Munich has research agreements with Elekta, Brainlab SE, and C‐RAD. Stefanie Corradini and Vanessa da Silva Mendes received speaker honoraria/travel support from Brainlab SE.

## FUNDING INFORMATION

Funding for research with Brainlab ExacTrac Dynamic was received from Brainlab SE. Open access funding enabled and organized by Brainlab SE.

Brainlab SE was not involved and had no influence on the study design, the collection, analysis or interpretation of data, on the writing of the manuscript or the decision to submit the manuscript for publication.

## ETHICS APPROVAL AND CONSENT TO PARTICIPATE

The selected patients were recruited in a prospective study (Ethics protocol 20–0664), which was approved by the local ethics committee of the University Hospital, LMU Munich. Written informed consent was obtained from all participants.
